# Identification and functional characterization of NbMLP28, a novel MLP-like protein 28 enhancing *Potato virus Y* resistance in *Nicotiana benthamiana*

**DOI:** 10.1186/s12866-020-01725-7

**Published:** 2020-03-06

**Authors:** Liyun Song, Jie Wang, Haiyan Jia, Ali Kamran, Yuanxia Qin, Yingjie Liu, Kaiqiang Hao, Fei Han, Chaoqun Zhang, Bin Li, Yongliang Li, Lili Shen, Fenglong Wang, Yuanhua Wu, Jinguang Yang

**Affiliations:** 1 College of Plant Protection, Shenyang Agricultural University, Shenyang, 110866 China; 2 Key Laboratory of Tobacco Pest Monitoring, Controlling & Integrated Management, Tobacco Research Institute of Chinese Academy of Agricultural Sciences, Qingdao, 266101 China; 3 Graduate School of Chinese Academy of Agricultural Sciences, Beijing, 100081 China; 4 Department of Science and Technology, State Tobacco Monopoly Bureau, Beijing, 100045 China; 5 Jiangxi Tobacco Research Institute, Nanchang, 330025 China; 6 Sichuan Tobacco Company, Chengdu, 610000 China; 7 Baoshan Company of Yunnan Tobacco Company, Baoshan, 678000 China

**Keywords:** *Potato virus* Y, Resistance, *N. Benthamian*, MLP-like proteins, NbMLP28, Jasmonic acid, Overexpression, VIGS, Gene silencing

## Abstract

**Background:**

Major latex proteins (MLPs) belong to the MLP subfamily in Bet v 1 protein family and respond to both biotic and abiotic stresses, which play critical roles in plant disease resistance. As the type species of widely distributed and economically devastating Potyvirus, *Potato virus Y* (PVY) is one of the major constraints to important crop plants including tobacco (*Nicotiana benthamiana*) worldwide. Despite the great losses owing to PVY infection in tobacco, there is no previous study investigating the potential role of MLPs in developing resistance to viral infection.

**Results:**

In this study, for the first time we have identified and functionally analyzed the MLP-like protein 28 from *N. benthamiana*, denoted as NbMLP28 and investigated its role in conferring resistance to *N. benthamiana* against PVY infection. NbMLP28 was localized to the plasmalemma and nucleus, with the highest level in the root. *NbMLP28* gene was hypothesized to be triggered by PVY infection and was highly expressed in jasmonic acid (JA) signaling pathway. Further validation was achieved through silencing of *NbMLP28* through virus-induced gene silencing (VIGS) that rendered *N. benthamiana* plants more vulnerable to PVY infection, contrary to overexpression that enhanced resistance.

**Conclusions:**

Taken together, this is the first study describing the role of NbMLP28 in tobacco against PVY infection and provide a pivotal point towards obtaining pathogen-resistant tobacco varieties through constructing new candidate genes of MLP subfamily.

## Background

*Potato virus Y* (PVY) is highly destructive plant virus with worldwide distribution and pose serious economic losses to tobacco production [1–3]. PVY is mainly transmitted systemically by aphids [4], and can lead to mosaic, mottle, dwarfism, deformity, and necrosis in tobacco plants, seriously damaging yield and quality [5]. Current control measures of PVY in tobacco rely heavily on aphid prevention, agronomic practices, and PVY-resistant tobacco varieties [6, 7]. To date, PVY-resistant tobacco varieties are rare, while the resistance of most of the tobacco germplasm is not achieved yet [8]. Plants employ multiple strategies to cope with virus infection. Such as, plant hormones trigger the defense response and enhance stress resistance upon infection [9]. Moreover, ethylene (ET), salicylic acid (SA), and jasmonic acid (JA) signaling participate in plant defense [10]. ET and JA cooperatively regulate induced systemic resistance (ISR) in plants in the presence of non-pathogenic microbes such as rhizobacteria. Ryu et al. reported that in Arabidopsis, JA induced by rhizobacterium could alleviate the symptoms caused by *Cucumber mosaic virus* (CMV) infection [11]. Furthermore, JA pretreatment followed by SA confers strong resistance against the *Tobacco mosaic virus* (TMV) in *N. benthamiana* [12].

The major latex protein (MLP) was first identified from the latex of opium poppy (*Papaver somniferum*) [13, 14]. MLP proteins are members of MLP subfamily in the Bet v 1 family and exist in many plant species [15] and the orthologues of MLP, the MLP-like proteins, are also found in various plant species including Arabidopsis, soybean and tobacco [16, 17]. Most of the MLP/RRP subfamily members in wild strawberry and cucumber were expressed during the fruit ripening [18, 19], and also by wounding in immature bell peppers [20]. As revealed by the microarray analyses of Arabidopsis, the expression of three paralogous MLP gene pairs was significantly downregulated upon oxidative stress, indicating that MLP may participate in stress response [21]. In addition, many studies have demonstrated the necessity of MLP function against pathogen infection. For instance, Arabidopsis *MLP28* (AT1G70830) and *MLP3* were induced by *Alternaria* and *Plasmodiophora brassicae,* respectively [22, 23], and MLP expression was detected in stem phloem sap of melon plants infected by CMV [24]. Despite the importance of MLPs in biotic and abiotic stress responses, no systematic study on the relationship between MLP family members and PVY infection has been conducted.

In this study, for the first time we have identified and cloned the MLP-like protein 28 (NbMLP28) gene from *N. benthamiana*. The expression profile of this gene revealed that it was responsive to PVY infection and defense-related signaling molecules including ET, JA, and SA. Furthermore, virus-induced silencing of *NbMLP28* rendered *N. benthamiana* plants more susceptible to PVY infection, whereas transient and constitutive overexpression of *NbMLP28* enhanced resistance in tobacco plants against PVY. In addition, we identified the pathway that modulates the expression of *NbMLP28* in *N. benthamiana*. The promoter sequence of *NbMLP28* was amplified and analyzed to contain cis-acting elements in response to JA, light, drought, auxin, endosperm expression, etc. Conclusively, this is the first identification of NbMLP28 in tobacco and also the first detailed study describing its importance as a contributor to plant defense against PVY infection and provide strong bases to obtain pathogen-resistant tobacco varieties through constructing new candidate genes of MLP subfamily.

## Results

### Identification of the *NbMLP28* gene and Phylogenetic analysis

**Fig. 1:**
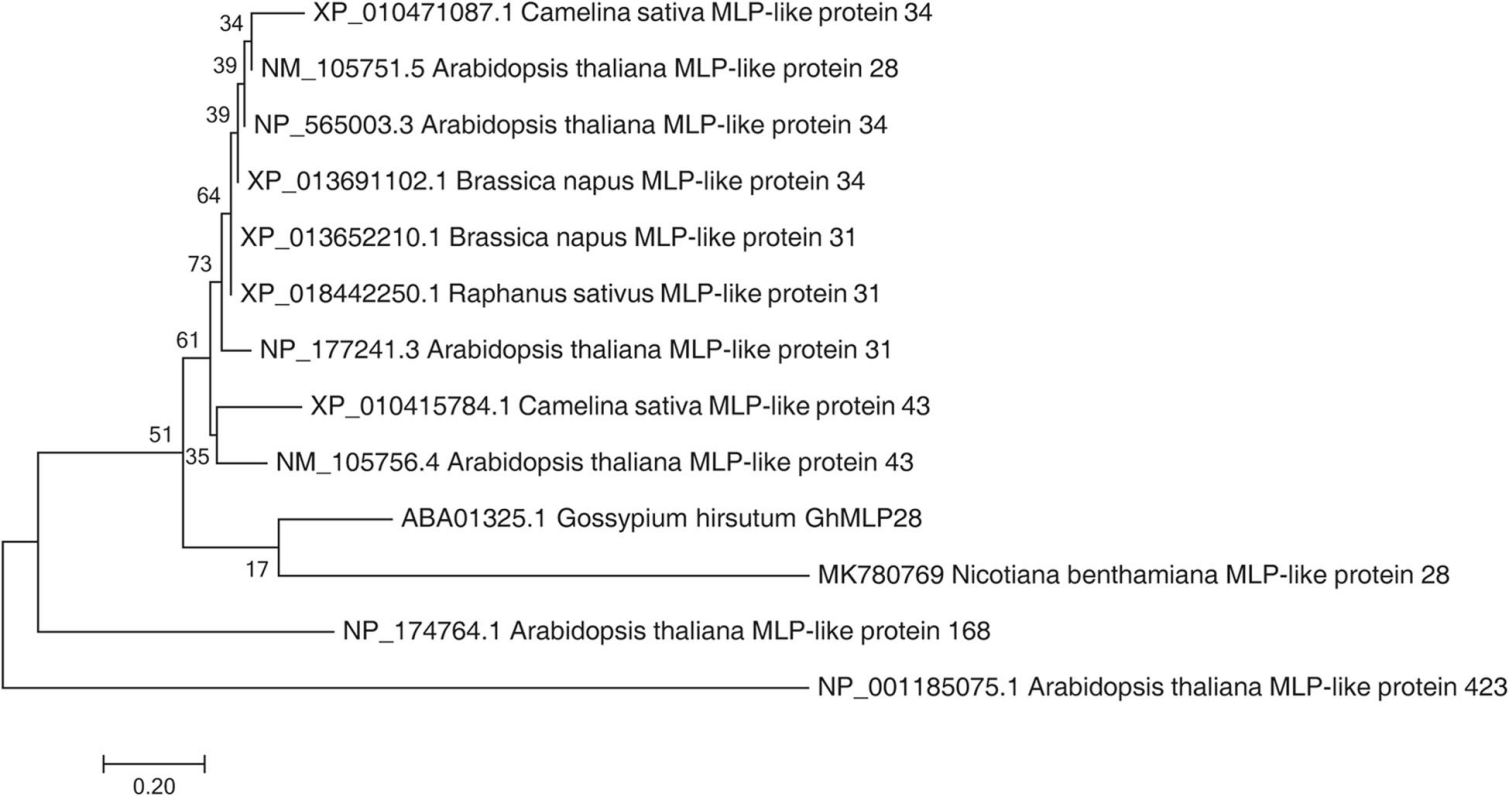
Phylogenetic analysis between MLP-like protein 28 of *Nicotiana benthamiana* and MLPs in other plants

**Fig. 2:**
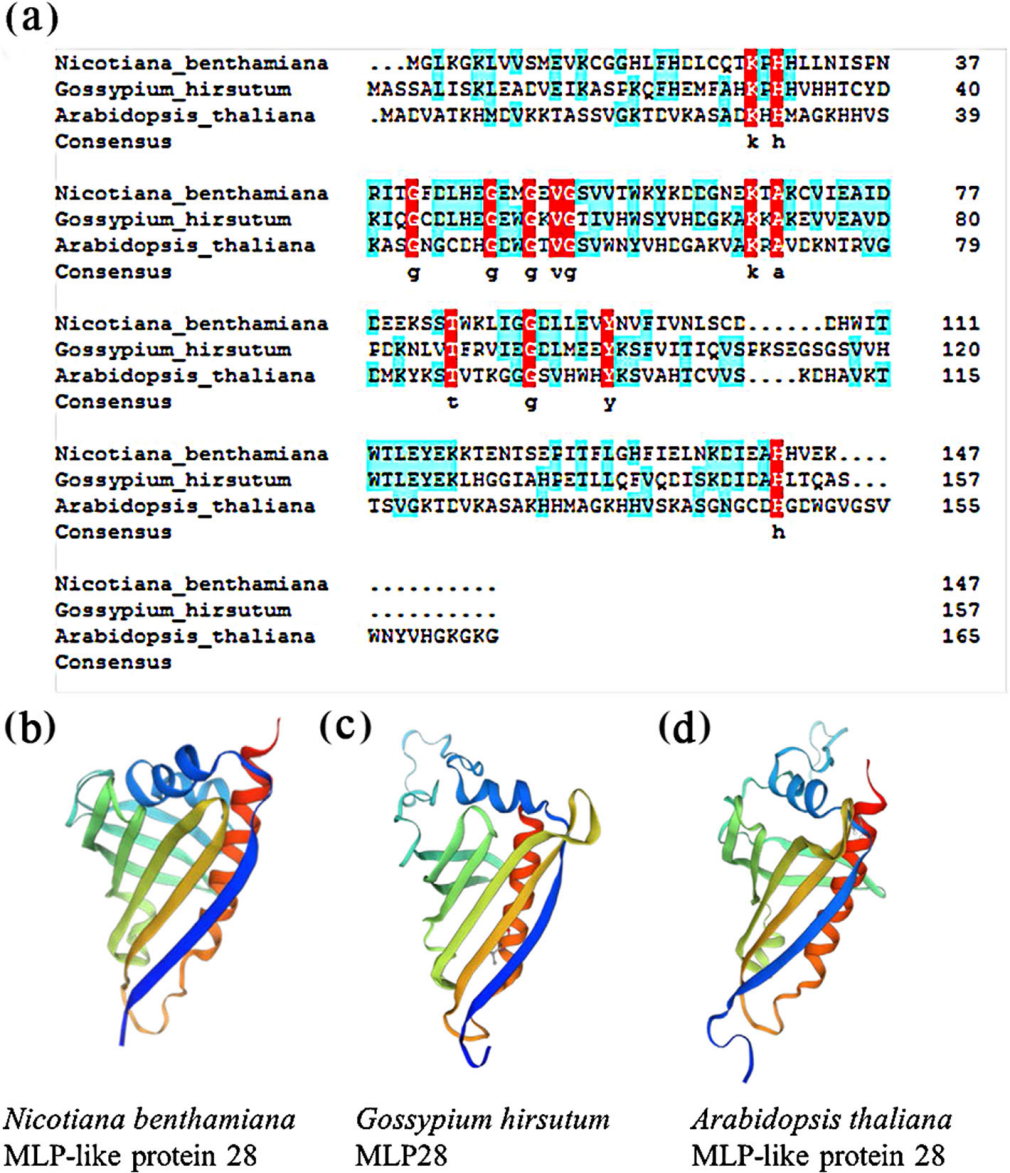
Sequence analysis of *N. benthamiana* MLP-like protein28. **a** Sequence alignment analysis of *N. benthamiana* MLP-like protein28 with *Arabidopsis thaliana* MLP28 (NM_105751.5) and *Gossypium hirsutum* MLP28 (ABA01325.1) using DNAMAN. **b**-**d** The 3D-structure of *N. benthamiana* MLP-like protein 28 with *Gossypium hirsutum* MLP28 and *Arabidopsis thaliana* MLP-like protein 28

We amplified the ORF of *NbMLP28* from *N. benthamiana* using primers, and the ORF of *NbMLP28* was aligned with the predicted ORF sequence of *NbMLP28* in the *N. benthamiana* database (https://solgenomics.net/organism/Nicotiana_benthamiana/genome). The ORF of *NbMLP28* was submitted to NCBI under accession number MK780769. We constructed a phylogenetic tree of *NbMLP28* and members of the MLP family in related species (Fig. 1). The result showed that the *NbMLP28* shares the highest sequence similarity with *Cossypium hirsutum* MLP28, which is a putative defense-related protein [25]. The multiple alignment analysis revealed 31.16% similarity between *Gossypium hirsutum* MLP28 and *Arabidopsis thaliana* MLP28, They all contain a Gly-rich loop whose sequence is GxxxxxG (Fig. 2a)*.* The structure of NbMLP28 protein predicted by SWISS-MODEL exhibits properties similar to those of the *Gossypium hirsutum* and *Arabidopsis* MLP28 (Fig. 2b-d). We cloned and analyzed the 3000 bp *NbMLP28* promoter region and identified several potential cis-acting elements involved in Me-JA and light responses, one MYB binding site involved in drought-inducibility, one auxin-responsive element, one element involved in the abscisic acid (ABA) response, and one enhancer-like element involved in anoxic-specific induction (Table 1).

**Table 1:** Cis-acting regulatory element analysis of the promoter of *NbMLP28* gene

Number	Site name	Amount	Sequence	Function of site
1	CGTCA-motif	2	CGTCA	cis-acting regulatory element involved in the MeJA-response
2	Gap-box	1	CAAATGAA(A/G)A	part of a light responsive element
3	I-box	1	GTATAAGGCC	part of a light responsive element
4	Box 4	3	ATTAAT	part of a conserved DNA module involved in light response
5	circadian	2	CAAAGATATC	cis-acting regulatory element involved in Circadian control
6	TGACG-motif	2	TGACG	cis-acting regulatory element involved in the MeJA-response
7	3-AF3 binding site	1	CACTATCTAAC	part of a conserved DNA module array (CMA3)
8	LAMP-element	1	CTTTATCA	part of a light responsive element
9	CCAAT-box	2	CAACGG	MYB Hv1 binding site
10	chs-CMA1a	1	TTACTTAA	part of a light responsive element
11	GCN4_motif	1	TGAGTCA	element involved in endosperm expression
12	CAAT-box	44	CCAAT	cis-acting element in promoter and enhancer regions
13	G-Box	1	CACGTT	regulatory element involved in light responsiveness
14	GATA-motif	3	AAGGATAAGG	part of a light responsive element
15	O2-site	2	GATGATGTGG	regulatory element involved in zein metabolism regulation
16	G-box	2	CACGTC	regulatory element involved in light responsiveness
17	MRE	1	AACCTAA	MYB binding site involved in light responsiveness
18	ARE	3	AAACCA	regulatory element essential for the anaerobic induction
29	MBS	1	CAACTG	MYB binding site involved in drought-inducibility
20	GC-motif	1	CCCCCG	enhancer-like element involved in anoxic specific inducibility
21	TGA-element	2	AACGAC	auxin-responsive element
22	ABRE	3	ACGTG	element involved in the abscisic acid responsiveness

### Subcellular localization of NbMLP28

**Fig. 3:**
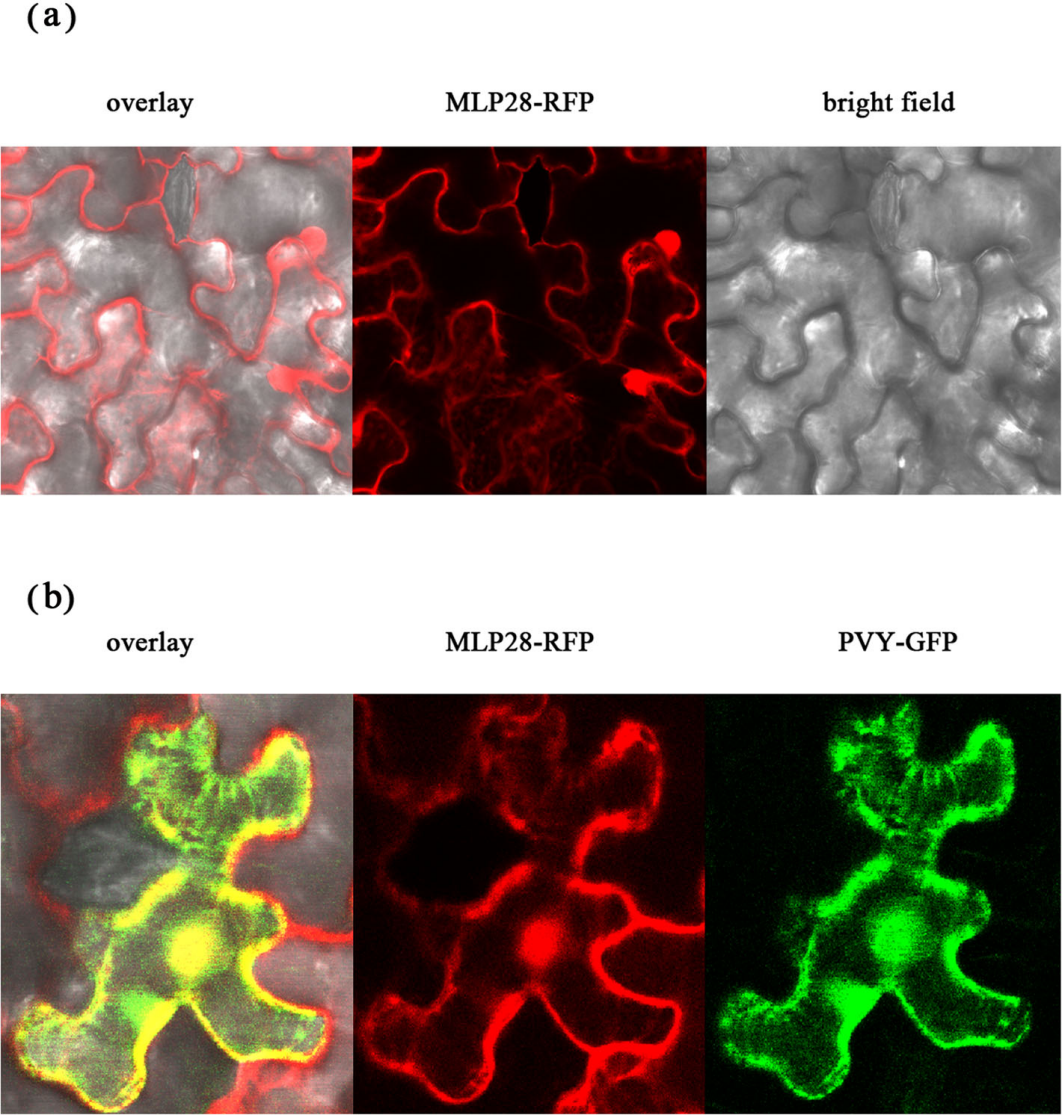
The subcellular localization of MLP-like protein 28. **a** The subcellular localization of MLP-like protein 28 in healthy *N. benthamiana*. **b** The subcellular localization of MLP-like protein during viral infection

We predicted the subcellular localization of NbMLP28 using an online Plant-mPLo tool (http://www.csbio.sjtu.edu.cn/bioinf/plant-multi/). The results suggested that the protein is localized in the cytoplasm. In addition, the protein contains a nuclear localization signal peptide (GLKGKLVVSMEVKCGGHLFHDLCQTKPHHLL) with a score of 4.2, as predicted by the NLS Mapper (http://nls-mapper.iab.keio.ac.jp/cgi-bin/NLS_Mapper_form.cgi#opennewwindow). Confocal results revealed that NbMLP28 is mainly localized to the plasmalemma and nucleus with or without virus infection (Fig. 3).

### Expression profiling of *NbMLP28*

**Fig. 4:**
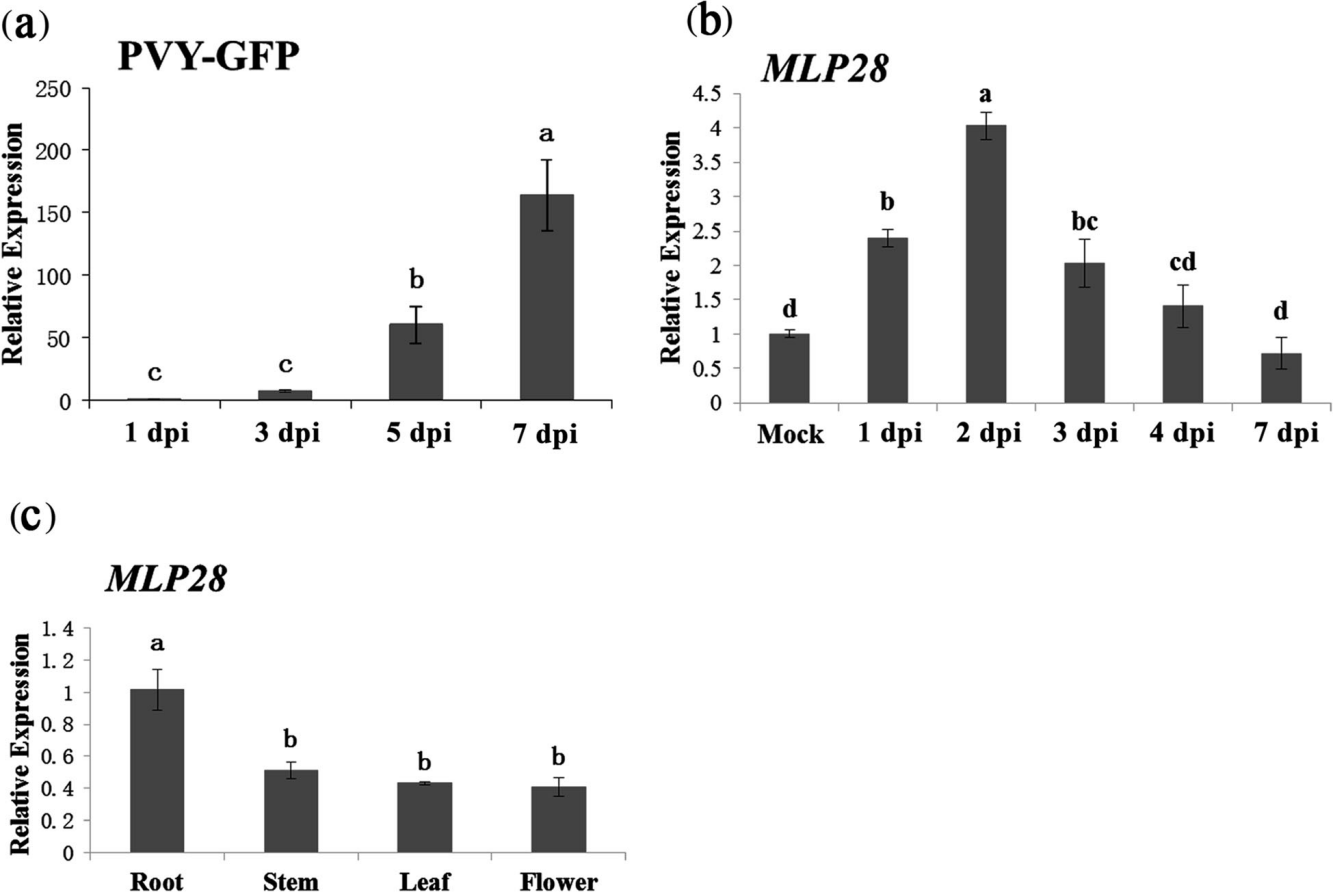
Gene expression pattern of *NbMLP28* in wildtype plants. **a** PVY-GFP expression trend at 1.3.5.7 days of inoculation. **b***NbMLP28* expression trend after PVY inoculation. **c***NbMLP28* expression trends in root, stem, leaf and flower of healthy plants. The data were analyzed by Duncan’s multiple range tests in the ANOVA program of SPSS, different letters indicate that values of the four treatments were significantly different at *P* < 0.05

The accumulation of virus showed an upward trend after 1, 3, 5, and 7 days post inoculation (dpi) of PVY, and reached the peak of at seven dpi (Fig. 4a). Likewise, the expression of *NbMLP28* was induced 1 day after PVY-GFP infection and maximized at 2 dpi (Fig. 4b). The qRT-PCR analysis detected uniformly *NbMLP28* expression in various tissues in healthy *N. benthamiana* plants and the root exhibited a relatively highest level of NbMLP28 transcripts than other tissues investigated (Fig. 4c).

### Silencing of *NbMLP28* Renders *N. benthamiana* plants more susceptible to PVY infection

**Fig. 5:**
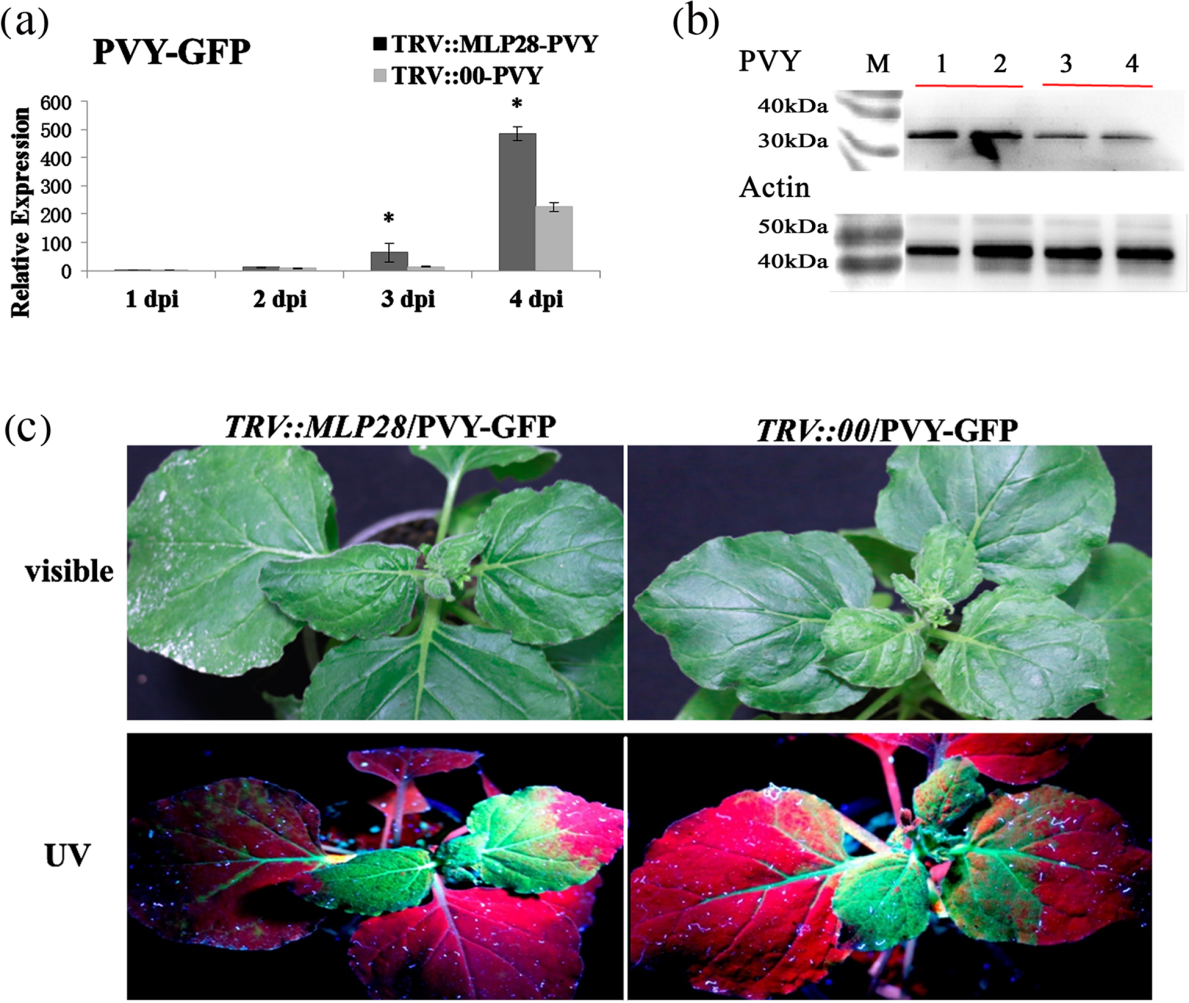
Effects of silencing *NbMLP28* on PVY infection. **a** PVY was inoculated after silencing *NbMLP28* 14 days, and the virus expression was higher compared to the control *TRV::00* inoculated PVY at the RNA level. **b** Samples inoculated with PVY for 3 days were used to detect viral protein expression differences by Western blotting. 1–2 is *TRV::MLP28* inoculated with PVY, 3–4 is *TRV::00* inoculated with PVY. **c** The fluorescence difference of these two treatments

To further investigate the role of *NbMLP28* in plant defense, we silenced the *NbMLP28* gene using VIGS. Silencing efficiency was tested by comparing the expression levels of *NbMLP28* in TRV::MLP28 plants versus TRV::00 control plants. Efficiency of the VIGS of *NbMLP28* was 87% and no phenotypic difference was observed between the *TRV::MLP28* and control plants (Supplementary Figure 1). Next, we infected the *TRV::MLP28* and control plants with PVY-GFP and monitored virus infection for at least 1 week. The results showed that virus infection in *TRV::MLP28* was significantly higher than that in the *TRV::00* control group one to 4 days following inoculation, the treatment was 3.6 times and 1.2 times higher than the control at 3 and 4 dpi, respectively. (Fig. 5a). Western blotting detected a relatively higher level of viral coat protein in *TRV::MLP28* than in *TRV::00* 3 days after virus inoculation (Fig. 5b, Supplementary Figure 4). Consistently, strong and far-ranging GFP signal was observed in *TRV::MLP28* leaves, whereas only fewer fluorescent spots were observed in *TRV::00* leaves. Especially, GFP signal was detected throughout the whole *TRV::MLP28* plant nine dpi but was only observable in the leaf vein, petiole and lower leaves in *TRV::00* individuals. The number and size of the infected areas in the systematic leaves of *TRV::MLP28* were significantly greater than those of *TRV::00* (Fig. 5c). Moreover, the *TRV::MLP28* plants showed severe malformation of emerging leaves as compared to the control at nine dpi, indicating the severity of PVY in the absence of this protein (Fig. 5c). Taken together, these results indicated that the silencing of *NbMLP28* rendered *N. benthamiana* plants highly susceptible to PVY infection.

### Transient *NbMLP28* overexpression enhances PVY resistance

**Fig. 6:**
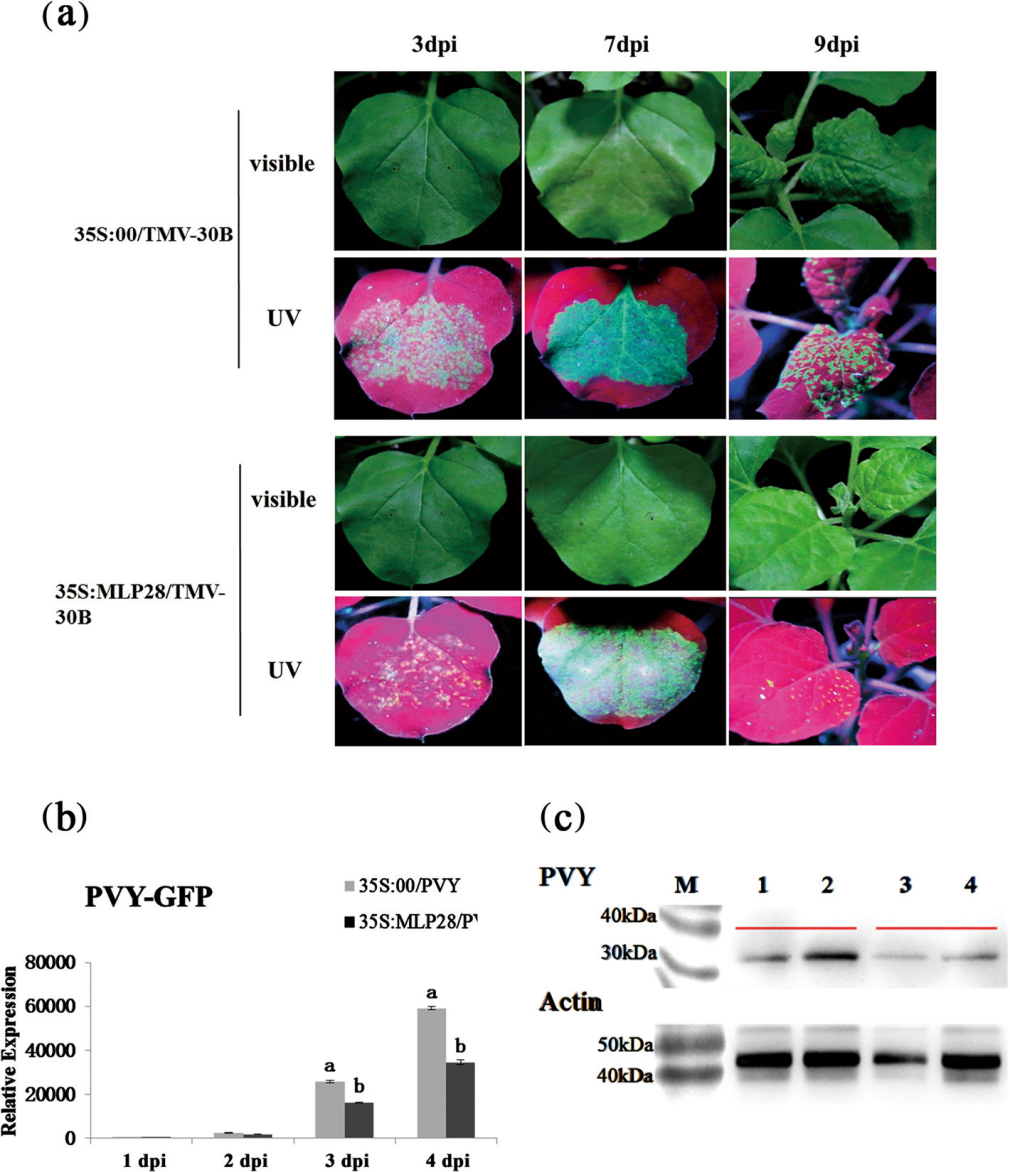
Effects of overexpression *NbMLP28* on PVY infection. **a** Transiently infiltrating tobacco *35::MLP28*/PVY-GFP and *35::00*/PVY-GFP, respectively, observed UV fluorescence difference at 3, 7 and 9 days of treatment. **b** Real-time PCR detected virus expression difference at 1, 2, 3, 4 days after inoculation. *35S::MLP28*/PVY was the treatment, *35S::00*/PVY was the control group. The data were analyzed with independent sample T test using SPSS Statistics v.21 software, different letters indicated that values of the two treatments were significantly different at *P* < 0.05. **c** Virus expression was differentially detected 3 days after PVY inoculation. 1–2 is *35S::00* inoculated with PVY, 3–4 is *35S::MLP28* inoculated with PVY

*NbMLP28* was transiently overexpressed in *N. benthamiana* to further determine its role in response to PVY infection. *N. benthamiana* leaves were infiltrated with *Agrobacterium* carrying the *35S::MLP28* construct or the empty vector *35S::00* (negative control). The differences in viral accumulation between PVY-GFP-infected *35S::MLP28* and *35S::00* leaves were assessed by examining the intensity of GFP signals. We continued to observe virus fluorescence differences of inoculated leaves and system leaves at 3dpi, 7dpi and 9dpi. Lower PVY accumulation was observed in *35S::MLP28* plants compared with the *35S::00* control three, seven, and 9 days after inoculation (Fig. 6a). For example, the number and size of infected areas in the systematic leaves of *35S::MLP28* were significantly reduced compared with those of the empty vector control 9 days after inoculation (Fig. 6a). There was no significant malformation of systematic leaves in *35S::MLP28* plants comparing with the control at 9 dpi (Fig. 6a). Moreover, the results of subsequent qRT-PCR and western blotting analyses were in line with the severity of PVY infection. Specifically, the PVY level in *35S::MLP28* leaves was reduced approximately 37% at 3 dpi and 42% at 4 dpi than *35S::00* leaves (Fig. 6b), and western blotting confirmed the lower PVY protein level in *35S::MLP28* than in *35S::00* at 3 dpi (Fig. 6c, Supplementary Figure 5). We also verified the results using *35S::MLP28* transgenic plants (Supplementary Figure 2). Taken together, these results supported the notion that *NbMLP28* overexpression enhanced PVY tolerance in *N. benthamiana* plants.

### *NbMLP28* overexpression in *N. benthamiana* promotes germination and root growth

**Fig. 7:**
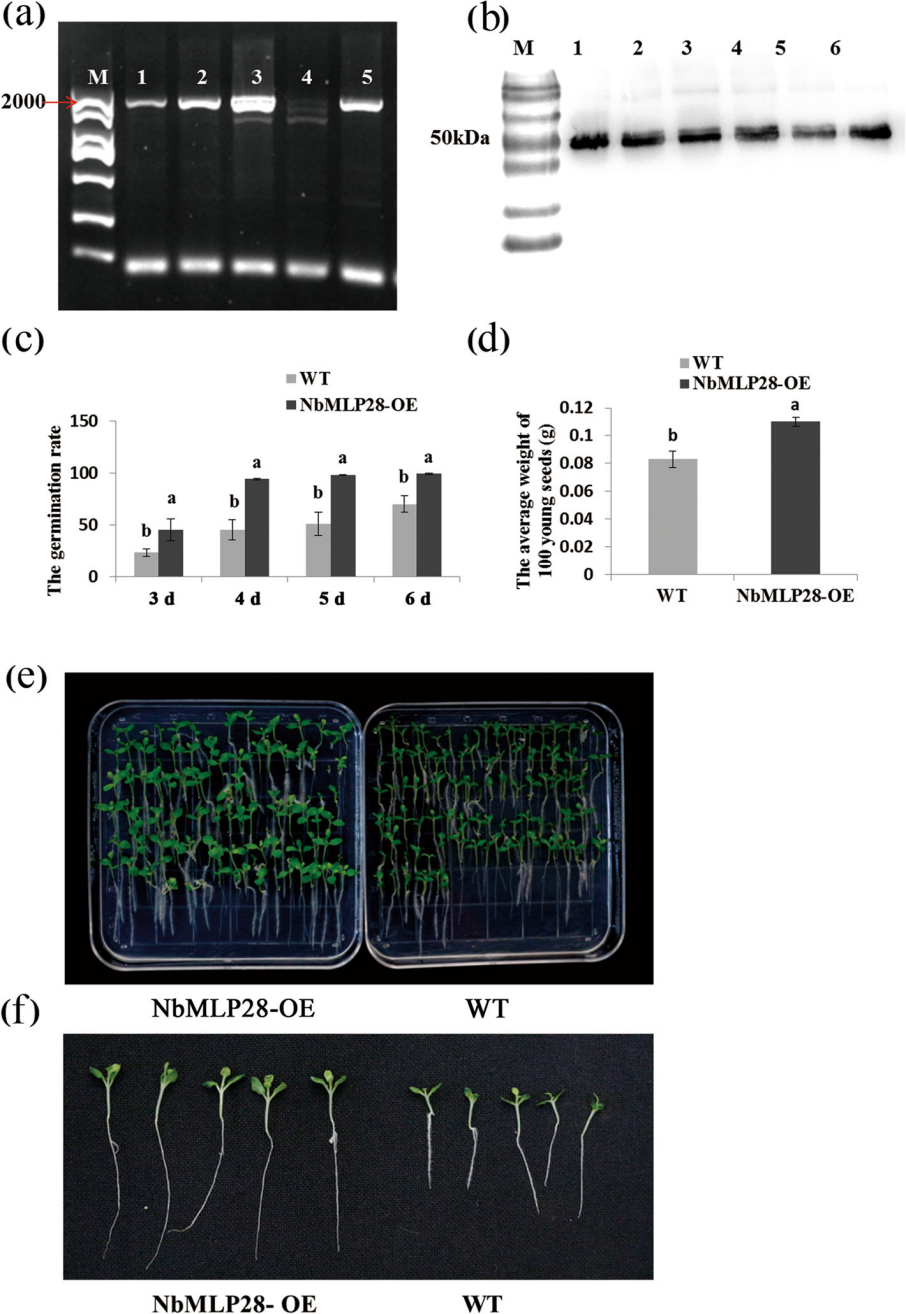
Overexpression of *NbMLP28* in transgenic *N. benthamiana* has germination and rooting promoting effect. **a** PCR amplification to selected highly overexpressed transgenic plants, sample 3 was a transgenic tobacco that strongly expresses *NbMLP28.***b** Protein detection of overexpressing plants T4 generation stably expressing RFP-tagged MLP28. **c** Statistics on germination rate of over-expressed *NbMLP28* transgenic tobacco and wild type at 3, 4, 5, and 6 days after seeding. **d** The fresh weight statistics of over-expression of *NbMLP28* transgenic tobacco and wild-type at 12 days after seeding. **e** Differences in growth status of overexpressed *NbMLP28* transgenic tobacco and wild type at 12 days after seeding. **f** Detection root length of over-expressed *NbMLP28* transgenic tobacco and wild-type at 12 days after seeding

Next, MLP28 was constitutively expressed in frame with a RFP tag under the 35S promoter (*35S::MLP28::RFP*) in wild-type *N. benthamiana*. Positive *35S::MLP28::RFP* transgenic plants were screened by PCR using primers E100F and E100R (Fig. 7a, Supplementary Figure 6). Through the antibiotic screening test, we obtained the T3 generation homozygous transgenic seeds and were used for subsequent experiments. The T_4_ plants of *35S::MLP28::RFP* were confirmed by western blotting using RFP antibody (Fig. 7b, Supplementary Figure 6). The *35S::MLP28::RFP* seeds started to germinate 2 days after sowing when the wild-type control showed no sign of germination. On day three, 45% germination of the *35S::MLP28::RFP* seeds was achieved, whereas only 23% of the wild-type seeds germinated. The roots of 12-day-old *35S::MLP28::RFP* plants were significantly longer than those of the wild-type seedlings and the two lines differed substantially in root morphology (Fig. 7e). The root length of the *35S::MLP28::RFP* plants averaged 2.8 cm, and the root length of the wild type control averaged 2.0 cm (Fig. 7f). Detailed statistical analyses revealed significant difference in germination rate between the *35S::MLP28::RFP* and control seeds three to 6 days after sowing (Fig. 7c). In addition, the fresh weight of *35S::MLP28::RFP* seedlings was significantly higher than that of the wild-type control (Fig. 7d). Collectively, these data revealed that *MLP28* overexpression promotes seed germination and root growth in tobacco.

### *NbMLP28* is highly responsive to JA signaling in *N. benthamiana*

**Fig. 8:**
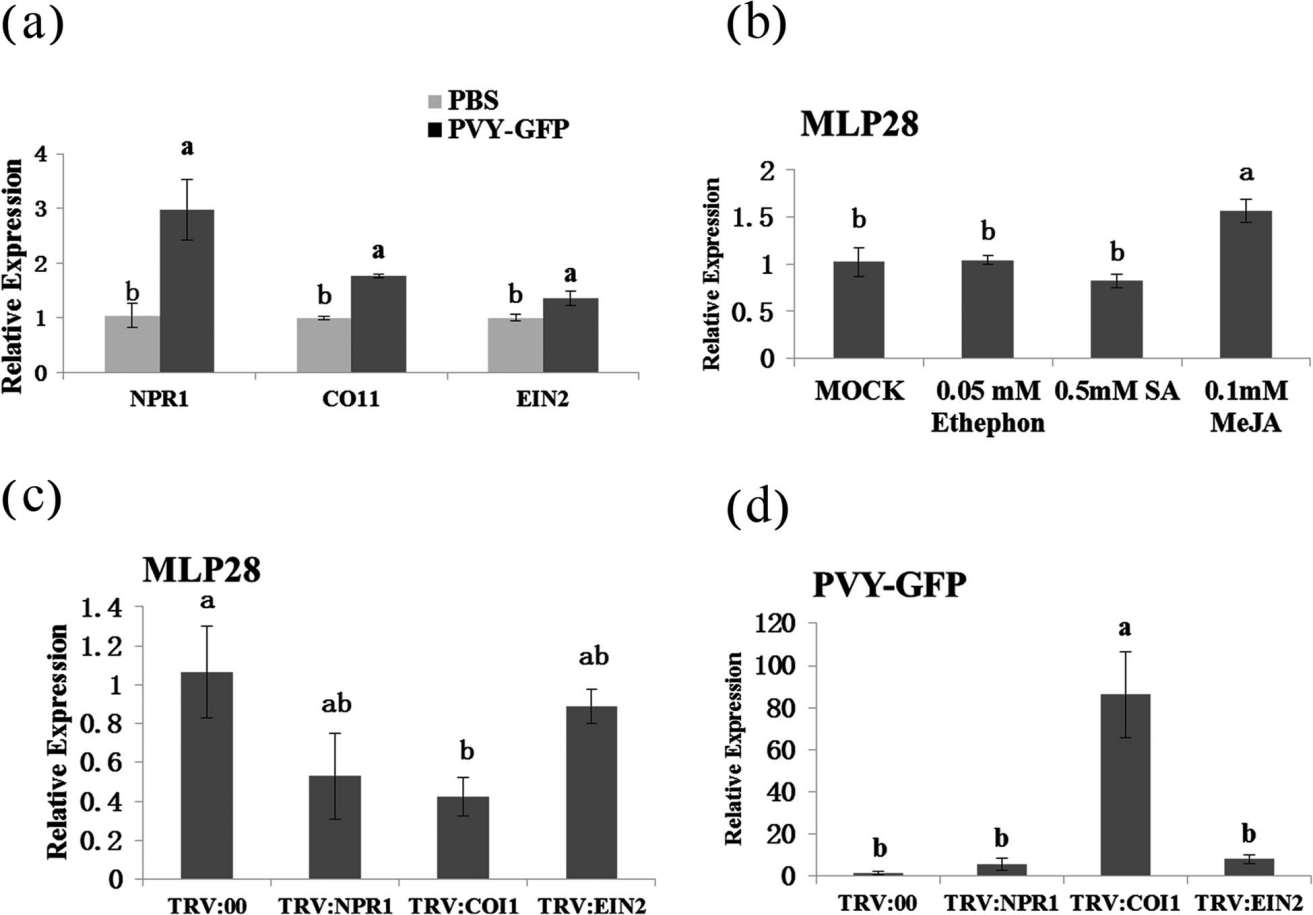
Identification of *NbMLP28* response to the hormonal pathway in *N. benthamiana*. **a** Expression of signal transduction key genes *NPR1*, *COI1* and *EIN2* of SA, JA and ET after 3 days of virus infection. The data were analyzed with independent sample T test using SPSS Statistics v.21 software, different letters indicate that values of the two treatments were significantly different at *P* < 0.05 (**b**) The expression of *NbMLP28* after spraying with 0.5 mM SA, 0.1 mM Me JA or 0.05 mM Ethephon, the MOCK was sprayed with water with 0.02% Tween 20. The data were analyzed by Duncan’s multiple range tests in the ANOVA program of SPSS, different letters indicate that values of the four treatments were significantly different at *P* < 0.05, the same as C and D. **c** The expression of *NbMLP28* after silencing *NPR1*, *COI1* and *EIN2*. **d** The expression of PVY-GFP after silencing *NPR1*, *COI1* and *EIN2*

To explore the molecular basis of *NbMLP28* in conferring PVY tolerance, we measured the relative expression levels of *NPR1*, *COI1* and *EIN2*, key genes in SA, JA and ET signaling, respectively, in 4-week-old *N. benthamiana* following PVY-GFP infiltration. Our data show that the expression levels of *NPR1*, *COI1* and *EIN2* were significantly influenced upon PVY-GFP infiltration (Fig. 8a), which is consistent with a previous finding that the SA, JA, and ET signaling pathways are involved in plant pathogens resistance [26]. To test the effects of SA, JA and ET on *NbMLP28* expression, we measured NbMLP28 transcript levels in *N. benthamiana* plants treated with 0.5 mM SA, 0.1 mM Me-JA or 0.05 mM Ethephon. Notably, Me-JA treatment significantly boosted the transcript level of *NbMLP28* by approximately 0.5-fold in *N. benthamiana* leaves (Fig. 8b). SA treatment slightly decreased the transcript level of *NbMLP28* by 0.2-fold in *N. benthamiana* plants, and 0.05 mM Ethephon had a minor effect on *NbMLP28* expression (Fig. 8b).

We then silenced the *NPR1*, *COI1* or *EIN2* genes in wild-type *N. benthamiana* plants using VIGS to further dissect the interactions between *NbMLP28* expression and SA, JA, and ET signaling [27].

The silencing efficiencies of *NPR1*, *COI1* and *EIN2* were 72, 70 and 75%, respectively (Supplementary Figure 7). We examined the expression pattern of *NbMLP28* under these three treatments and observed a strong *NbMLP28* reduction upon *NPR1* and *COI1* silencing—relative *NbMLP28* expression was down-regulated by 50 and 60% in *TRV::NPR1* and *TRV::COI1* individuals than *TRV::00*, respectively. By contrast, *EIN2* silencing only slightly reduced *NbMLP28* expression by 16% (Fig. 8c). Meanwhile, boosted PVY-GFP expression was observed in *TRV::NPR1*, *TRV::COI1* and *TRV::EIN2* transgenic lines compared with that of the control group, with *TRV::COI1* individuals showing the highest level of PVY-GFP expression (Fig. 8d). Taken together, these findings suggested the responsiveness of *NbMLP28* to JA signaling.

## Discussion

Despite the importance of MLP proteins in biotic and abiotic stress responses, their role in PVY-tobacco interaction remain unclear. In this study, we identified NbMLP28, a novel MLP-like protein 28 and investigated its functional profile in response to PVY infection in *N. benthamiana*. *NbMLP28* was localized in both, the plasmalemma and nucleus. It was expressed uniformly in tobacco plants with the root exhibiting the highest level. Furthermore, the expression level of *NbMLP28* peaked 2 days after PVY infection in tobacco plants. Whilst, transient and stable transgenic plants overexpressing *NbMLP28* were more resistant to PVY infection, whereas silencing of this gene facilitated the viral infection.

The isolated ORF of *MLP28* from *N. benthamiana* encoded a protein of 147 amino acids with a predicted conserved Bet v 1 domain, which was named after Bet v 1, a ribonuclease-active birch pollen allergen PR-10 protein [28]. PR-10 accumulation could be induced by pathogen infection, abiotic stress, related signaling molecules, hypersensitive response (HR), and systemic acquired resistance (SAR) [29], which was important for plant’s defense against biotic and abiotic stresses [30]. The presence of this Bet v 1 domain in NbMLP28 strongly indicated that it might be involved in plant defense. An MLP gene (At4g14060) has been reported to be down-regulated to a significant level during infection of Arabidopsis by *Plum pox virus* (PPV) – like PVY, an important member of the genus *Potyvirus* [31]. While, MLP induction has been a common observation following *Verticillium dahliae* (*V. dahlia*) attack in cotton [32–34], and ectopic overexpression of *GhMLP28* in tobacco leads to improved *V. dahliae* tolerance [35]. In line with these reports, we have also observed an enhanced PVY resistance at both the mRNA and protein levels in *N. benthamiana* overexpressing *NbMLP28*.

Plant hormones are known to play essential roles in biotic and abiotic stress responses. Previous studies have identified the Arabidopsis MLP43 as a positive regulator of drought response, which modulated water loss efficiency, electrolyte leakage, ROS levels, and the expression levels of genes involved in ABA signaling [36], cotton MLP28 induced ethylene responsive factor 6 upon *V. dahlia* infection [35]. In accordance with these findings, our results showed that the NbMLP28 at transcriptomic level significantly elevated in tobacco after the exogenous application of Me-JA, as compared to SA and ET. We concluded that *NbMLP28* responds to PVY infection via the JA signaling pathway. Additionally, the silencing of *NPR1*, *COI1* and *EIN2*, the key genes involved in hormone signaling, downregulated *NbMLP28* expression in *N. benthamiana*, where silencing of *COI1* significantly decreased the *NbMLP28* expression and enhanced PVY accumulation as compared to *NPR1*and *EIN2*. Similar findings have indicated that *NPR1* and *COI1* silencing in tobacco led to increased TMV susceptibility [12], suggesting that reduced *NbMLP28* expression in *COI1*-silenced plants might hampered systemic resistance in *N. benthamiana* against PVY infection. Meanwhile, through analyzing the 3000 bp *NbMLP28* promoter sequence, we identified two cis-regulatory elements (with the CGTCA and TGACG motifs, respectively) involved in Me-JA-response. However, the detailed mechanism by which *NbMLP28* induced these responses still require further studies.

No phenotypic difference was observed between the *35S::MLP28* and control plants, indicating that *NbMLP28* overexpression did not affect the growth and development of transgenic tobacco plants (Supplementary Figure 3). Conclusively, the higher *NbMLP28* expression level is associated with increased PVY resistance in *N. benthamiana.* To our knowledge, this is the first mechanistic study of how NbMLP28 modulates the resistance of *N. benthamiana* against PVY infection. However, the detailed molecular mechanism by which this protein affected the defense pathway warrant future research.

## Conclusions

This is the first ever identification and functional analysis of NbMLP28 in PVY-infected *N. benthamiana*. Additionally, we have analysed its defensive role upon PVY infection, that will further provide strong bases for constructing new candidate genes of MLP family to develop disease-resistant varieties of tobacco.

## Methods

### Plant materials

Two sets of *N. benthamiana* plants were used: (a) wild-type (seeds were obtained from Key Laboratory of Tobacco Pest Monitoring, Controlling & Integrated Management, Tobacco Research Institute)*,* (b) *NbMLP28* overexpressing transgenic plants (constructed in current study) were grown in a growth chamber with 50–60% humidity and a 16 h/8 h light/dark photoperiod at 25 °C. For inoculation, PVY-GFP (obtained from Key Laboratory of Tobacco Pest Monitoring, Controlling & Integrated Management, Tobacco Research Institute) was used in this study.

### Cloning and sequence analysis of *NbMLP28*

RNA was isolated from *N. benthamiana* leaves using the TRIzol reagent (Vazyme) and first-strand cDNA synthesis was carried out using 2 μg total RNA and 100 U reverse transcriptase (Vazyme). Gene-specific primers MLP28F and MLP28R (Supplementary Table 1) were designed based on *N. benthamiana* genome data of Sol Genomics Network and used for PCR amplification; the resulting amplicons were subjected to 1% agarose gel electrophoresis and Sanger-sequenced. The deduced amino acid sequences of MLP28 (designated NbMLP28) were aligned with orthologs in other species in DNAMAN and SWISS-MODEL was employed for structure prediction [37]. The phylogenetic tree was generated using MEGA7 [38]. The potential cis-regulatory elements within *NbMLP28* promoter were analyzed using the online program Plant CARE (http://bioinformatics.psb.ugent.be/webtools/plantcare/html/).

### Virus-induced gene silencing (VIGS)

TRV vectors were kindly provided by Dr. Yule Liu, Tsinghua University, Beijing, China. Preparation of the pTRV vectors and *Agrobacterium tumefaciens* for VIGS followed a previously described procedure [39]. For VIGS vector construction, a 200 bp partial coding sequence (CDS) of *NbMLP28* was amplified from a cDNA library of *N. benthamiana* leaf using gene-specific primers MLP28-TRVF and MLP28-TRVR (Supplementary Table 1) and inserted into the pTRV2 vector. For the VIGS assay, pTRV1 or pTRV2 constructs harboring the *NbMLP28* fragment were introduced into the *Agrobacterium* strain LBA4404. Equal amount of *Agrobacterium* cultures containing pTRV1 and pTRV2 or pTRV2-MLP28 was mixed and used to inoculate the lower leaves of four-leaf stage *N. benthamiana* plants using a 1-mL needleless syringe. To determine VIGS efficiency, the leaves of tobacco plants 14 days post-inoculation (dpi) were tested by qRT-PCR using primers MLP28 QF and MLP28 QR (Supplementary Table 1), which detected the sequence outside the targeting fragment on the pTRV2-MLP28. Positive silencing plants were selected 14 dpi for analyzing *NbMLP28* function. To test the response of *NbMLP28* to hormones, we employed the same method described above to silence key hormone signaling genes *NPR1*, *COI1* and *EIN2* from the SA, JA and ET signaling pathways, respectively, and compared the expression levels of *NbMLP28* and PVY-GFP in *N. benthamiana*.

### Vector construction and agrobacterium-mediated gene transformation

To overexpress *NbMLP28*, the CDS of *NbMLP28* was amplified from *N. benthamiana* with primers MLP28-35SF and MLP28-35SR, which contain the *Xba*I and *Eco*RI restriction sites, respectively (Supplementary Table 1). The resulting PCR fragment was inserted between the restriction sites on the Fu46-RFP entry vector. The target fragment was then inserted into the pEarlyGate100 expression vector that contains the 35S promoter, the resulting construct was introduced into the *A. tumefaciens* strain LBA4404 using a freeze–thaw method. *A. tumefaciens* cultures carrying *35S::MLP28::RFP* were incubated overnight at 28 °C, harvested the next morning, and resuspended and cultured in an infiltration buffer containing 10 mM MES (pH = 5.6), 10 mM MgCl_2_, and 150 μM acetosyringone until OD600 reached 0.8. After three-hour incubation at room temperature, the bacterial suspensions were used to infiltrate the lower leaves of *N. benthamiana* plants using a needleless syringe for transient overexpression experiments.

We also overexpressed *NbMLP28* in wild-type *N. benthamiana* using the same overexpression construct. First, a 5–8 mm disc was taken from a sterile tobacco leaf using a puncher, and the disc was placed on a preculture medium, and cultured at 25 °C for 24 h under light for 18 h. Then, the Agrobacterium of the vector was suspended in a liquid co-cultivation medium, the OD value was adjusted to 0.5–1.0, and the explants were inoculated for 30 min. The explants were placed on the co-culture medium and cultured at 24 °C for 3 days under light for 18 days. After the completion of the co-cultivation, the explants were transferred to a selection medium, cultured at 28 °C, 18 h light, and subcultured once every 2 weeks. In the selection medium, the explants grew longer and the buds grew from the callus. When the bud point grows to a length of 3 mm, it was transferred to the rooting medium. The tobacco plants were moved to the culture soil after about 2 weeks. Positive seedlings were detected with primers E100F/E100R, and the seeds were subcultured. We disinfected the surface of the T3 seeds and placed it on one-half MS medium of 50 mg/L Kan. After 1 week, the seedlings were all green, indicating that homozygous transgenic seeds had been obtained. In addition, tobacco leaves that overexpress NbMLP28 were infected with PVY-GFP after confirming the expression of *35S::MLP28* by PCR and western blotting analysis.

### GFP and RFP imaging

The subcellular localization of NbMLP28 was examined using a Leica SP8 confocal microscope (Leica Microsystems, Shanghai) 48 h after the transient expression of *NbMLP28* with a RFP tag in *N. benthamiana* epidermal cells. The plants were grown under a 16 h/8 h light/dark cycle at 25 °C. For the subcellular localization experiment, GFP was excited with a 25 mW, 488 nm argon laser, and emitted light with a wavelength between 495 and 535 nm was captured; RFP was excited with a 25 mW, 552 nm argon laser, and emitted light with a wavelength between 580 and 630 nm was captured. Successive images of 20 μm × 20 μm were scanned sequentially using 488 nm and 552 nm lasers with a 1.0 s scanning interval [40]. For the *NbMLP28* silencing and overexpression experiments, in order to visually detect the accumulation of virus in inoculated leaves, we infiltrated the tobacco leaves with PVY-GFP and observed the difference in fluorescence between the treated and the control under a hand-held UV lamp (Ultra-Violet Products, Upland, CA, USA). One inoculated leaf per plant was measured and three biological replicates were analyzed for each line.

### Hormone treatment

Four-week-old wild-type *N. benthamiana* seedlings were grown in a growth chamber under conditions mentioned above. The leaves were sprayed with 0.5 mM SA, 0.1 mM Me-JA, or 0.05 mM Ethephon with 0.02% Tween 20. The control plants were sprayed with water and 0.02% Tween 20. Three biological replicates of the wild-type *N. benthamiana* were analyzed. The treated leaves were harvested 24 h after the treatments, immediately snap frozen in liquid nitrogen and stored at − 80 °C until use.

### Quantitative real-time PCR

Total RNAs isolation and cDNA synthesis followed the same procedures described above. qRT-PCR was performed with the SYBR Premix Ex Taq™ kit (Vazyme) using the Applied Biosystems 7500 Fast Real-Time PCR system (Applied Biosystems, Waltham, MA, USA) following the manufacturers’ instructions. The *β-Actin* gene was used as the endogenous control. *NbMLP28* and *β-Actin* were amplified using primer pairs MLP28 QF/MLP28 QF and β-Actin QF/β-Actin QR, respectively (Supplementary Table 1). Meanwhile, two PVY primers, PVY-F and PVY-R, were used to detect the changes in virus coat protein expression. The − 2^-△△CT^ method was used to calculate the relative expression level of target gene and three biological replicates were analyzed for each line [41].

### Western blotting

For western blotting, protein was isolated from *N. benthamiana*, and total plant proteins were 1:1 equal volume mixed with 2 × SDS-PAGE buffer. Next, the protein samples were incubated at 95 °C for three min and separated on a 12% SDS-polyacrylamide gel. The separated proteins were then transferred onto nitrocellulose membranes by electroblotting instrument. The PVY CP antibody (SRA20001, Agdia, USA), anti-RFP (ab62341, Abcam, Shanghai) and β-Actin (CW0264M, CWBIO, Beijing) antibody were used for this assay.

### Morphological characterization of the transgenic plants

Seeds of the wild-type and *NbMLP28::RFP* overexpression *N. benthamiana* lines received in the same batch were surface-sterilized and sown on one-half Murashige & Skoog plates. The plates were stratified at 4 °C for 24 h and let grow vertically at 25 °C with a 16 h/8 h light/dark photoperiod to examine root morphology. Plant root of the plants was measured over a one-week period. These experiments were repeated three times with 100 plants of each of the WT and *NbMLP28::RFP* lines were used per replicate.

### Statistical analysis

Mean values of at least three independent experiments are shown and standard deviations (S.D.) are given. Duncan’s multiple range test analysis of variance (ANOVA), and independent sample *t*-test were performed in SPSS (v.21, IBM, Armonk, NY, USA). *P* < 0.05 denotes significant differences between comparisons.

## Supplementary information

**Additional file 1: Table S1.** The primers used in this paper. **Figure S1.** The efficiency of agrobacterium-mediated virus-induced gene silencing in *N. benthamiana*. (A) Photographs were taken at 14 days after TRV infiltration. *TRV::00* is a negative control, *TRV::PDS* as a positive control. Experiments were repeated three times with similar results. (B) Silencing efficiency between treatment and control was detected using real-time PCR. **Figure S2.** Differences in virus expression between overexpressing *NbMLP28* transgenic plants and wild type at 7 days of PVY-GFP inoculation. (A) Fluorescence differences in overexpression of *NbMLP28* transgenic plants and wild type at 7 days of PVY-GFP inoculation. (B) Differences in RNA levels between overexpressing *NbMLP28* transgenic plants and wild type at 7 days of PVY-GFP inoculation. (C) Differences in viral protein between overexpressing *NbMLP28* transgenic plants and wild type at 7 days of PVY-GFP inoculation. **Figure S3.** The phenotype of *35S::MLP28::RFP* transgenic *N. benthamiana* and wild-type at 2-week old seedlings and 4-week old seedlings. **Figure S4.** The original western blotting figure of PVY CP differences respective in silencing and transient overexpression *NbMLP28*. (A) The first four lanes are *35S::00* and *35S::MLP28*, and the last four lanes are *TRV::MLP28* and *TRV::00*, Marker (14-120 kDa). (B) The Actin figure of corresponding samples, Marker (14-120 kDa). **Figure S5.** The original western blotting figure of wild-type and *35S::MLP28::RFP* transgenic plants in response to PVY stress. (A) The first and second lanes respective were differences of PCY CP when wild-type and transgenic plants were inoculated with PVY at 7 dpi, Marker (14-120 kDa). (B) The Actin figure of corresponding samples, Marker (14-120 kDa). **Figure S6.** The full length original images of Gel and blot, presented in Fig. 7. (A) The original map of PCR to detect *NbMLP28* highly expressed transgenic plants (We only selected the gel map of the first 5 lanes, showing the differences in expression of *NbMLP28* in different transgenic plants, so as to select strong expression of *NbMLP28* transgenic plants), Marker (DL2000, Vazyme). (B) The original map of validating the T4 generation stably expressing RFP tags in overexpressed plants, Marker (14-120 kDa). **Figure S7.** The silencing efficiency of *NPR1*, *COI1* and *EIN2* in *N. benthamiana*.

## Data Availability

Full length sequence of NbMLP28 was submitted in GenBank with the accession number: MK780769. All the raw data is publicly available and included in the manuscript also. Further details can be requested at jiayoulily2009@126.com.

## Abbreviations

*35S::00::RFP*: an empty vector carrying an RFP tag
*35S::MLP28::RFP*: an *MLP28* overexpression vector carrying an RFP tag
ABA: Abscisic acid
CMV: *Cucumber mosaic virus*
dpi: day post inoculation
ET: Ethylene
HR: Hypersensitive response
ISR: Induced systemic resistance
JA: Jasmonic acid
*NbMLP28*: MLP-like protein 28 gene in *N. benthamiana*
NbMLP28: MLP-like protein 28 in *N. benthamiana*
PPV: *Plum pox virus*
PVY CP: PVY capsid protein
PVY: *Potato virus Y*
PVY-GFP: PVY with a green fluorescent protein label
RFP: A red fluorescent protein
SA: Salicylic acid
SAR: Systemic acquired resistance
TMV: *Tobacco mosaic virus*
VIGS: Virus-Induced Gene Silencing
