# Supplementary material for: Identification and functional characterization of NbMLP28, a novel MLP-like protein 28 enhancing Potato virus Y resistance in Nicotiana benthamiana
Source: BMC Microbiol. 2020 Mar 6;20:55. doi: 10.1186/s12866-020-01725-7 (PMC7060652; doi:10.1186/s12866-020-01725-7)
Supplement: Supplementary file 1 — Additional file 1: Table S1. The primers used in this paper. Figure S1. The efficiency of agrobacterium-mediated virus-induced gene silencing in N. benthamiana. (A) Photographs were taken at 14 days after TRV infiltration. TRV::00 is a negative control, TRV::PDS as a positive control. Experiments were repeated three times with similar results. (B) Silencing efficiency between treatment and control was detected using real-time PCR. Figure S2. Differences in virus expression between overexpressing NbMLP28 transgenic plants and wild type at 7 days of PVY-GFP inoculation. (A) Fluorescence differences in overexpression of NbMLP28 transgenic plants and wild type at 7 days of PVY-GFP inoculation. (B) Differences in RNA levels between overexpressing NbMLP28 transgenic plants and wild type at 7 days of PVY-GFP inoculation. (C) Differences in viral protein between overexpressing NbMLP28 transgenic plants and wild type at 7 days of PVY-GFP inoculation. Figure S3. The phenotype of 35S::MLP28::RFP transgenic N. benthamiana and wild-type at 2-week old seedlings and 4-week old seedlings. Figure S4. The original western blotting figure of PVY CP differences respective in silencing and transient overexpression NbMLP28. (A) The first four lanes are 35S::00 and 35S::MLP28, and the last four lanes are TRV::MLP28 and TRV::00, Marker (14-120 kDa). (B) The Actin figure of corresponding samples, Marker (14-120 kDa). Figure S5. The original western blotting figure of wild-type and 35S::MLP28::RFP transgenic plants in response to PVY stress. (A) The first and second lanes respective were differences of PCY CP when wild-type and transgenic plants were inoculated with PVY at 7 dpi, Marker (14-120 kDa). (B) The Actin figure of corresponding samples, Marker (14-120 kDa). Figure S6. The full length original images of Gel and blot, presented in Fig. 7. (A) The original map of PCR to detect NbMLP28 highly expressed transgenic plants (We only selected the gel map of the first 5 lanes, showing the di [file 12866_2020_1725_MOESM1_ESM.docx]

Supplementary Table 1. The primers used in this paper

| Name | Primers |
| --- | --- |
| MLP28F | 5’- ATGGGTTTGAAAGGTAAATTGGTTGT -3’ |
| MLP28R | 5’- CTATTTTTCGACATGGTGAGCCTCA -3’ |
| MLP28 QF, qRT-PCR | 5’- GCACATGGAAACTGATAGGAGGAG-3’ |
| MLP28 QR, qRT-PCR | 5’- TCCAGTGGTCATCACATGATAGGT-3’ |
| β-Actin QF, qRT-PCR | 5’-CAAGGAAATCACCGCTTTGG-3’ |
| β-Actin QR, qRT-PCR | 5’-AAGGGATGCGAGGATGGA-3’ |
| MLP28 proF | 5’-AAGATTGGAGAGGTGGCTTATGAGCTTG-3’ |
| MLP28 proR | 5’-GTGCCGGAAATTGATTTTTTTATTG-3’ |
| MLP28-TRVF | 5’-TAAGGTTACCGAATTCATGGGTTTGAAAGGTAAATTGGT-3’ |
| MLP28-TRVR | 5’-AGACGCGTGAGCTCGGTACCTTTTCATTTCCATCATCCTTATATT-3’ |
| MLP28-35SF | 5’-CTTTAGATCTTCTAGAATGGGTTTGAAAGGTAAATTGGTTG-3’ |
| MLP28-35SR | 5’-AGGAGGCCATGAATTCTTTTTCGACATGGTGAGCCTC-3’ |
| E100F | 5’-CATCATTGCGATAAAGGAAAGGC -3’ |
| E100R | 5’-GGCGGTAAGGATCTGAGCTACAC -3’ |
| PVY-F | 5’-GATTTGCCTAAGGGTTGGTTTCG -3’ |
| PVY-R | 5’- GATGAATGGGCTTATGGTTTGGTG-3’ |
| NPR1 QF, qRT-PCR | 5’-ATCTCTTGCTATGGCAGGCGATG -3’ |
| NPR1 QR, qRT-PCR | 5’-ACCGTTGTCCTCTGTGCGTTG -3’ |
| COI1 QF, qRT-PCR | 5’-GCTCCACGCGATTACCAACGG -3’ |
| COI1 QR, qRT-PCR | 5’-CTGCCACCATCTCTTGCACACC -3’ |
| EIN2 QF, qRT-PCR | 5’-GTATGGAATTCAGGAGCGGAAGGC -3’ |
| EIN2 QR qRT-PCR | 5’-AGAAGACGGAAGCACAAGAGCAAC -3’ |
| NPR1-TRVF | 5’-TAAGGTTACCGAATTCATGGATAATAGTGGGACTGCGT -3’ |
| NPR1-TRVR | 5’-AGACGCGTGAGCTCGGTACCCGGACTCCTCGCCGACAA-3’ |
| COI1-TRVF | 5’-TAAGGTTACCGAATTCCTTGATAATGGTGTCCGTGC-3’ |
| COI1-TRVR | 5’-AGACGCGTGAGCTCGGTACCCCAACGTATCCCAGAAGCATC-3’ |
| EIN2- TRVF | 5’-TAAGGTTACCGAATTCATGGAATCTGAAACTCTGACTATAG-3’ |
| EIN2- TRVR | 5’-AGACGCGTGAGCTCGGTACCATATTCTTCACTGCAAATCTGGGC-3’ |

| 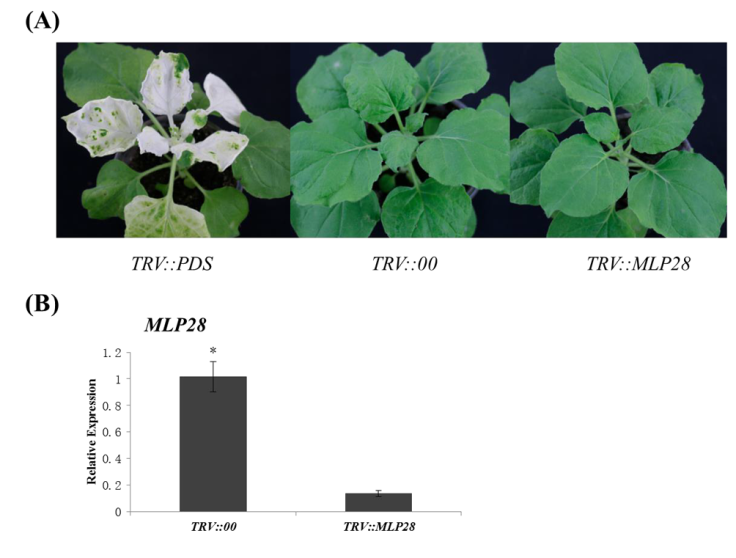 |
| --- |
| **Supplementary Figure 1.** The efficiency of agrobacterium-mediated virus-induced gene silencing in *N. benthamiana*. (A) Photographs were taken at 14 days after TRV infiltration. *TRV::00* is a negative control, *TRV::PDS* as a positive control. Experiments were repeated three times with similar results. (B) Silencing efficiency between treatment and control was detected using real-time PCR. |

| 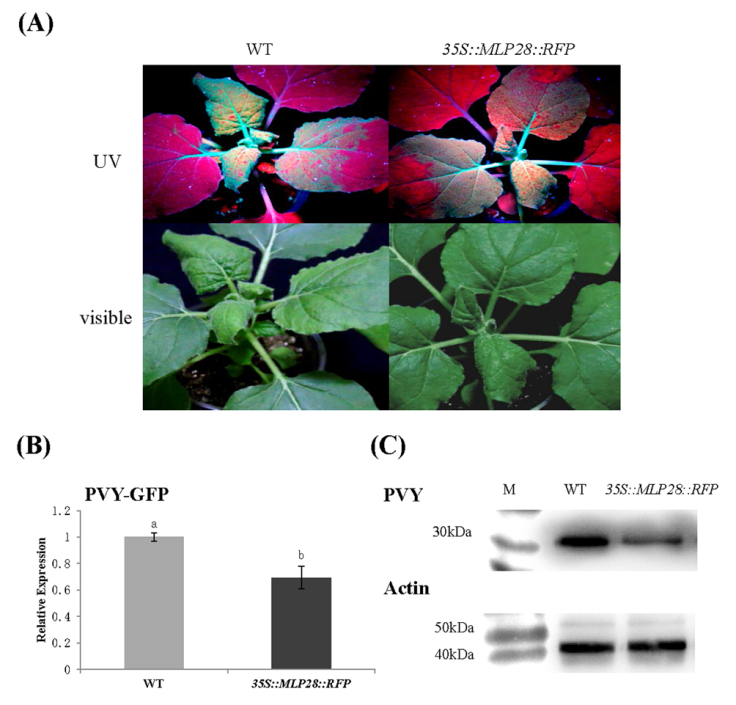 |
| --- |
| **Supplementary Figure 2.** Differences in virus expression between overexpressing *NbMLP28* transgenic plants and wild type at 7 days of PVY-GFP inoculation. (A) Fluorescence differences in overexpression of *NbMLP28* transgenic plants and wild type at 7 days of PVY-GFP inoculation. (B) Differences in RNA levels between overexpressing *NbMLP28* transgenic plants and wild type at 7 days of PVY-GFP inoculation. (C) Differences in viral protein between overexpressing *NbMLP28* transgenic plants and wild type at 7 days of PVY-GFP inoculation. |

| 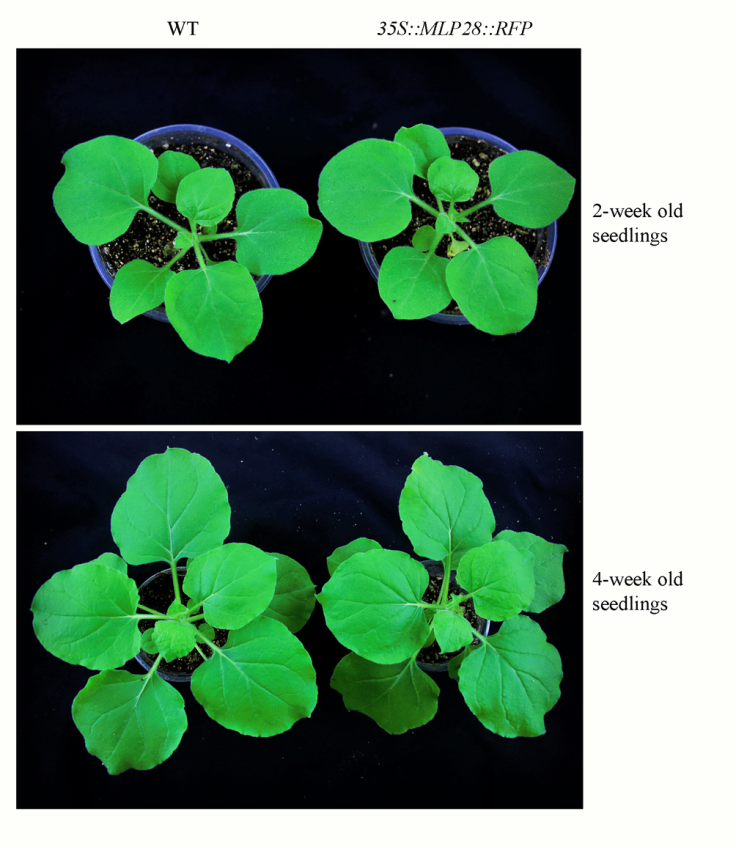  **Supplementary Figure 3.** The phenotype of *35S::MLP28::RFP* transgenic *N. benthamiana* and wild-type at 2-week old seedlings and 4-week old seedlings. |
| --- |

| 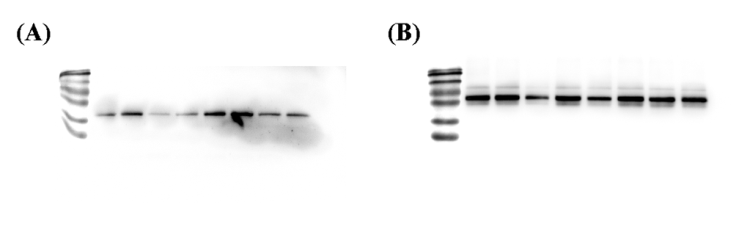 |
| --- |
| **Supplementary Figure 4.** The original western blotting figure of PVY CP differences respective in silencing and transient overexpression *NbMLP28*. (A) The first four lanes are *35S::00* and *35S::MLP28*, and the last four lanes are *TRV::MLP28* and *TRV::00*, Marker (14-120kDa). (B) The Actin figure of corresponding samples, Marker (14-120kDa). |

| 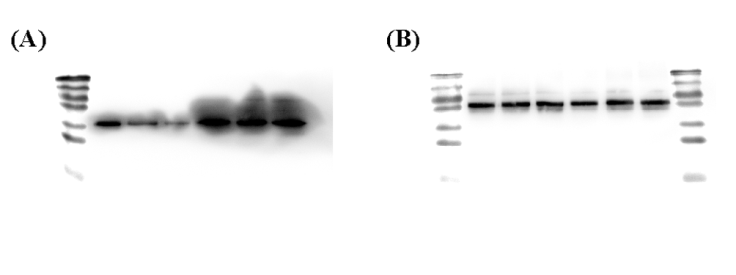 |
| --- |
| **Supplementary Figure 5.** The original western blotting figure of wild-type and *35S::MLP28::RFP* transgenic plants in response to PVY stress. (A) The first and second lanes respective were differences of PCY CP when wild-type and transgenic plants were inoculated with PVY at 7 dpi, Marker (14-120kDa). (B) The Actin figure of corresponding samples, Marker (14-120kDa). |

| 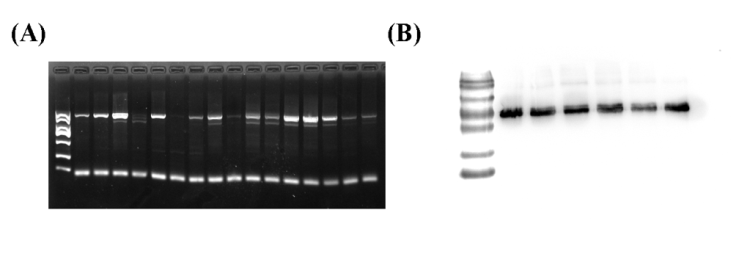 |
| --- |
| **Supplementary Figure 6.** The full length original images of Gel and blot, presented in Figure 7. (A) The original map of PCR to detect *NbMLP28* highly expressed transgenic plants (We only selected the gel map of the first 5 lanes, showing the differences in expression of *NbMLP28* in different transgenic plants, so as to select strong expression of *NbMLP28* transgenic plants), Marker (DL2000, Vazyme). (B) The original map of validating the T4 generation stably expressing RFP tags in overexpressed plants, Marker (14-120kDa). |

| 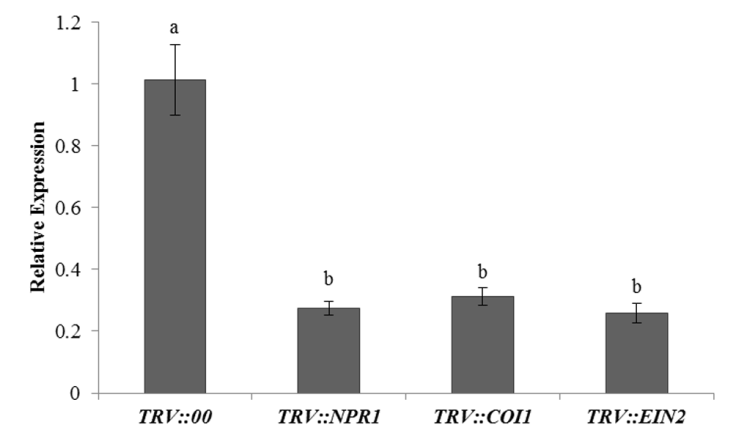 |
| --- |
| **Supplementary Figure 7.** The silencing efficiency of *NPR1*, *COI1* and *EIN2* in *N. benthamiana*. |
